# TNFα, IL-6, miR-103a-3p, miR-423-5p, miR-23a-3p, miR-15a-5p and miR-223-3p in the crevicular fluid of periodontopathic patients correlate with each other and at different stages of the disease

**DOI:** 10.1038/s41598-022-26421-6

**Published:** 2023-01-04

**Authors:** Erica Costantini, Bruna Sinjari, Pamela Di Giovanni, Lisa Aielli, Sergio Caputi, Raffaella Muraro, Giovanna Murmura, Marcella Reale

**Affiliations:** 1 Department of Medicine and Science of Aging, University “G. d’Annunzio”, 66100 Chieti, Italy; 2 Department of Innovative Technologies in Medicine and Dentistry, University “G. d’Annunzio”, 66100 Chieti, Italy; 3 Department of Pharmacy, University “G. d’Annunzio”, 66100 Chieti, Italy

**Keywords:** Molecular biology, Health care, Pathogenesis

## Abstract

Periodontitis is one of the main frequent intraoral diseases. Pathogenesis triggers are the immune responses with pro-inflammatory cytokines production and non-coding RNAs expression. The purpose of the present study was to evaluate the involvement of selected miRNAs in various stages of periodontitis and their relationship with the levels of inflammatory mediators in gingival crevicular fluid (GCF). For this study, 36 subjects (21 with periodontal disease, 15 healthy controls) were selected with an age mean of 59.1 ± 3.7 years. Clinical parameters included plaque index, gingival index, sulcus bleeding index, pocket depth, and clinical attachment level. The GCF samples were taken using capillary paper. The levels of miRNAs in GCF were estimated using a Real-Time PCR and TNFα and IL-6 levels were assessed by enzyme-linked immunosorbent assay (ELISA). The results indicated that the miRNA-103a-3p, miRNA-23a-3p, miRNA-15a-5p, and miRNA-223-3p were significantly upregulated with respect to healthy controls. Significant differences were observed for miRNA-23a-3p, miRNA-103a-3p and miRNA-423-5p levels in accord with the disease stages. Inflammatory mediators evaluated in GCF correlate well with the clinical parameters and the severity of the periodontal disease. miRNAs can represent biomarkers of disease stage and can be investigated as a possible therapeutic target, as well as levels of TNFα and IL-6 may drive the disease progression by acting as prognostic markers.

## Introduction

The frequent physical and mechanical stress of tissue in the periodontal region and the highly dynamic remodeling determines the development of periodontitis, one of the main chronic inflammatory diseases affecting more than 50% of the world population^1^. Periodontitis is influenced by the interplay between subgingival microbial dysbiosis, periodontal tissue inflammation, tissue destruction, and genetic alterations, with an imbalanced host response^2^. A new periodontitis classification developed by a consensus conference between American Academy of Periodontology (AAP) and European Academy of Periodontology (EFP) has been adopted, providing an assessment of disease grading (A,B,C) and staging (I, II, III, IV). Staging depends on the severity of the disease at presentation and its management complexity, while grading provides additional information on the biological characteristics of the disease, including an analysis of periodontitis progression, based on patient history (Tables 1, 2)^3^.

**Table 1:** Periodontal staging according to the new classification of AAP/EFP^3^.

Periodontal staging		Stage I	Stage II	Stage III	Stage IV
Severity	Interdental at the sites of greatest bone loss	1–2 mm	3–4 mm	≥ 5 mm	≥ 5 mm
	Radiographic bone loss	Coronal third (< 15%)	Coronal third (15–33%)	Extending to the mid-third of root and beyond	Extending to mid- third of root and beyond
	Tooth loss	No	No	Tooth loss due to periodontitis ≤ 4	Tooth loss due to periodontitis ≥ 5
Complexity	Local	MPD ≤ 4 mm	MPD ≤ 5 mm	In addition to stage II complexity MPD ≥ 6 Vertical bone loss ≥ 3 Furcation involved class II/III Moderate ridge defect	In addition to stage III complexity

MPD, mean probing depth.

**Table 2:** Periodontal grading according to the new classification of AAP/EFP^3^.

Periodontal grading			Grade A: slow rate of progression	Grade B: moderate rate of progression	Grade C: rapid rate of progression
Primary criteria	Direct evidence of progression	Longitudinal data (radiographic bone loss or CAL)	Evidence of no loss over 5 years	< 2 mm over 5 years	> 2 mm over 5 years
	Indirect evidence of progression	%bone loss/age	< 0.25	0.25–1	> 1
		Case phenotype	Heavy biofilm deposits with low levels of destruction	Destruction commensurate with biofilm deposits	Destruction exceeds expectation given biofilm deposits; specific clinical patterns of rapid progression and/or early onset disease
Grade modifiers	Risk factors	Smoking	Non-smoker	Smoker ≤ 10 cigarettes	Smoker ≥ 10 cigarettes/day
		Diabetes	Normoglycemic/ no diagnosis of diabetes	HbA1c < 7%	HbA1c > 7%

The stage I represents the early stages of attachment loss; the stage II represents established periodontitis in which periodontal examinations can identify damages at the tooth support. Stage III of periodontitis produces significant damage to the attachment apparatus and tooth loss may occur, in the absence of advanced treatment; this stage is characterized by the presence of deep periodontal lesions and presence of deep intra-bony defects. Stage IV causes significant damage to periodontal support and may cause significant tooth loss, resulting in loss of masticatory function^4^.

The relationship between immune system and periodontal disease has been highlighted in the regulation of the pathogenetic mechanism of periodontitis, both in the initial stages and in the progression toward a chronic condition. The pathogens responsible for the initiation of periodontal disease recall the mediators of innate immunity, neutrophils, and macrophages at the damage site, producing cytokines and other inflammatory products^5^. When there is no resolution of the lesion, the specific immune response is activated with the intervention of lymphocytes, macrophages, and dendritic cells. The majority of literature confirm that neutrophils are hyperactive in periodontitis, in particular in severe, early onset forms, at the same time investigators have observed reduced neutrophil functions^6^. The dysregulation of inflammation and immune pathways, responsible for tissue damage, progression and chronicization of periodontal disease, is highly linked to genetic and epigenetic alterations.

Being epigenetic modulators, the microRNAs (miRNAs), short sequences (19–24 nucleotides in length) of non-coding RNAs, interact with the 3′ untranslated regions (3′ UTR) of target messenger RNAs (mRNA) causing mRNA degradation, translational suppression^6^ and interfere with the post-transcriptional expression of multiple target genes playing an important regulator role of the immune response. Indeed, miRNAs are responsible for neutrophils’ activity control and their migration to the inflammatory site, as they are involved in the regulation of mRNA sequence stability as well as the inflammatory mediator’s production^7,8^.

In vivo and in vitro studies have defined the miRNAs as determinants of periodontitis’ pathogenesis, as promoters of microbial persistence and as deregulators of the innate and adaptive immune response which become ineffective against pathogens and lead to the worst prognosis of the disease^8–10^. Moreover, miRNAs expression is also related to osteogenesis and osteoclastogenesis, mainly related to osteoclasts’ activity in bone-resorption and the osteoblasts’ proliferation and differentiation, both necessary for bone tissue homeostasis^11–13^.

Accumulating evidence suggests that the differential expression of miRNAs in gingival tissues and fluids can be causally linked to periodontitis and be promising candidates as potential disease biomarkers^14,15^.

Based on their characteristics in terms of expression stability and profiles, observed in serum and plasma, saliva and more recently also in the gingival crevicular fluid (GCF), miRNAs are emerging as tools for the detection of alterations of the oral cavity and of systemic diseases^14,15^. These biofluids can be easily and quickly collected with a minimally invasive procedure, bestowing great potential for the diagnostic and prognostic value of periodontal disease.

Recent investigations on inflammatory response and bone tissue homeostasis have reported a significant increase in salivary expression levels of miR-146a, miR-155 and miR-223, inhibiting osteoclastogenesis and driving the nuclear factor kappa-light-chain-enhancer of activated B cells (NF-kB) pathway activation, in patients affected by periodontitis^16–18^. Moreover, in chronic periodontitis, it has been confirmed a relationship between salivary miRNAs expression and periodontitis' pathological processes, with the highlighting of the contribution of miRNAs (miR-142-3p, miR-146a, miR-155, miR-203, and miR-223) involved in the regulation of bacterial infections, inflammation, and immune response^16,19,20^.

MiR-223, in particular, has been reported as key regulator of the innate immune responses mostly in association with the ability of the myeloid lineage differentiation^21^, inflammatory response modulation and infection development^22^.

Similarities were reported between miR-223 and miRNAs 15 and 23 based on inflammatory target pathways regulation^23,24^.

Among the miRNAs overexpressed during inflammation, the miR-103 is reported to target tumor necrosis factor (TNF)α, interleukin (IL)-17, IL-1β and the pathway NF-kB^25^. These miRNAs are also related to the osteoclast development process and bone metabolism, such as the miRNA 423^26^.

Furthermore, literature evidence suggests an association of miRNAs with inflammatory cytokines such as IL-1α, IL-1β, TNFα, IL-6 and IL-8, which are known to play an active role in periodontal tissue disease and whose unregulated production appears to be involved in chronic leukocyte recruitment and promoting tissue destruction^5^.

Thus, the aim of this study was to explore for the first time the involvement of miR-15a-5p, miR-23a-3p, miR223-3p, miR-103a-3p, miR-423-5p and TNFα, IL-6 production in periodontal disease pathological and healthy GCF.

## Results

### Demographic and periodontal parameters

The demographic characteristics of the enrolled subjects are summarized in Table 3. The mean age of participants was not significantly different in the periodontal disease group in comparison with the healthy controls (HC). Also, gender distribution, Body Mass Index (BMI), and lifestyle habits were not statistically different within and between the groups.

**Table 3:** Demographic characteristics of periodontitis and HC groups.

Variable	Periodontitis (*n* = 21)	HC (*n* = 15)
sex, *n (%)*		
Male	14 (67)	11 (74)
Female	7 (33)	4 (26)
Age (years), *mean* ± *SD*	60.4 ± 3.0	58.5 ± 3.5
BMI (kg/m^2^), *mean* ± *SD*	24.2 ± 3.6	22.9 ± 2.7
**Smoking habits, *n (%)***		
Yes	3 (14.3)	9 (60)
No	18 (85.7)	6 (40)
**Alcohol intake**		
Yes	6 (28.6)	9 (60)
No	15 (71.4)	6 (40)
**Diet**		
Omnivore	21 (100)	12 (80)
Vegetarian	0 (0)	3 (20)

Data are expressed as the mean ± standard deviation (SD) or number (n) and percentage (%).

BMI, body mass index.

As the more accurate indicators of the healthy periodontal support structure around a tooth, clinical attachment level (CAL) and maximum probing depth (MPD) have been used to define the disease stage. The clinical attachment level was measured with probe from cemento-enamel junction (CEJ) to the bottom of periodontal pocket. The maximum probing depth was measured from the gingival margin to the bottom of the gingival sulcus/ pocket. As shown in Table 4, patients were grouped into 3 disease stages: mild which correspond to grade II (8 patients), moderate as grade III (6 patients) and severe as grade IV of new periodontitis classification (7 patients).

**Table 4:** Oral clinical parameters of the periodontitis group.

	Mild (n = 8)	Moderate (n = 6)	Severe (n = 7)
CAL 12–21 (mm)	4 ± 0.5	5 ± 0.4	7 ± 0.9
CAL 31–41 (mm)	4 ± 0.5	5 ± 0.9	7 ± 1.0
CAL 36 (mm)	4 ± 0.6	6 ± 0.7	6 ± 1.2
CAL 46 (mm)	4 ± 0.6	6 ± 0.9	6 ± 1.2
CAL 26 (mm)	4 ± 0.7	6 ± 1.1	6 ± 1.4
CAL 16 (mm)	4 ± 0.9	5 ± 0.1	5 ± 1.0
MPD (mm)	5.1 ± 1.1	7.4 ± 1.6	7.6 ± 1.5

Data summarized as mean $\pm$ SD.

CAL, clinical attachment level; MPD, maximum probing depth.

### TNFα and IL-6 quantification in periodontitis and HC groups

To define the inflammatory condition in patients with periodontitis, TNFα and IL-6 levels were quantified by ELISA assay in patients’ GCF. As reported in Table 5, a significant and progressive increase was observed in relation to the disease stage. Significant higher levels of both cytokines were observed in periodontitis patients at mild, moderate and severe stages with respect to HC (p < 0.001), underlying the involvement of these inflammatory cytokines in the pathogenesis and progression of periodontitis.

**Table 5:** Levels of TNFα and IL-6.

Variable	TNFα (pg/ml)	IL-6 (pg/ml)
Mild periodontitis	24.14 (± 0.64)	117.51 (± 7.24)
Moderate periodontitis	30.22 (± 0.23)	161.93 (± 6.70)
Severe periodontitis	32.33 (± 1.32)	278.81 (± 1.94)
HC	19.19 (± 0.24)	70.95 (± 0.91)
*p-value*	< 0.001	< 0.001

Data are reported as mean ± SD. The statistically significant difference between different periodontitis patients’ stages and HC (Student t-test, p ≤ 0.001). Data were analyzed using Stata® version 15.

### miRNAs expression levels in GCF in periodontitis and HC groups

**Figure 1:**
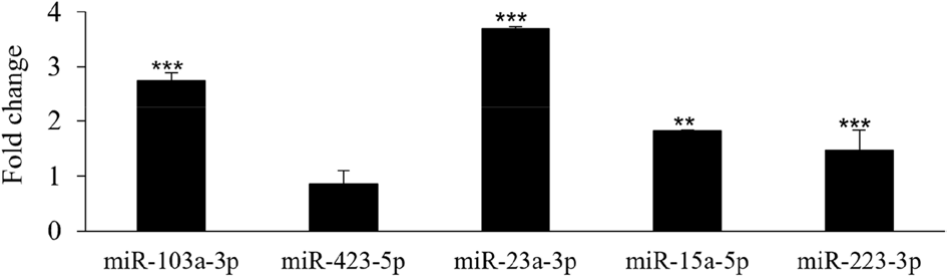
Relative expression levels of miRNAs detected in GCF of patients with periodontitis compared to HC. Data are reported as fold change ± C.I. with respect to HC, equal to 1. The statistical significances shown are **p < 0.01; ***p < 0.001. Data were analyzed using Stata version 15 and GraphPad Prism 6.

The expression levels of miRNAs 103a-3p, miR423-5p, miR23a-3p, miR15a-5p, and miR223-3p in periodontitis patients and HC were analyzed by Real-time PCR. Figure 1 shows that miR103a-3p, miR23a-3p, miR15a-5p, and miR223-3p were significantly upregulated in GCF collected in the periodontitis group in comparison with the HC, while miR423-5p showed no difference in fold change.

### miRNAs expression levels in periodontitis patients with different disease stages

**Figure 2:**
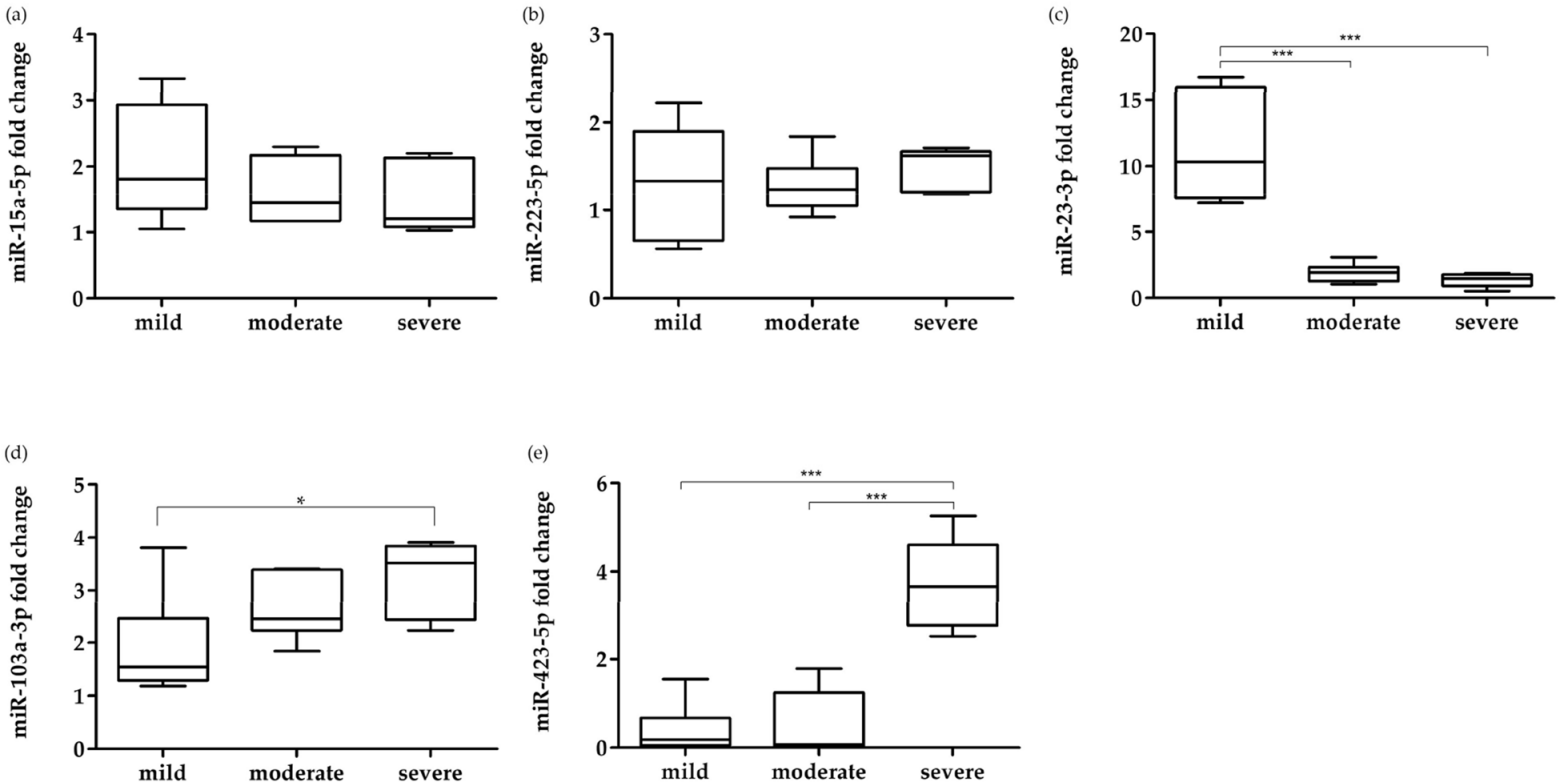
Boxplots of relative expression levels of (**a**) miR-15a-5p, (**b**) miR-223-3p, (**c**) miR-23a-3p, (**d**) miR-103a-3p and (**e**) miR-423-5p in periodontal disease patients GCF sample, at mild, moderate and severe disease stages. Data are reported as fold change ± C.I. with respect to HC, equal to 1. The statistical significances shown are *p < 0.05; **p < 0.01; ***p < 0.001. Data were analyzed using Stata version 15 and GraphPad Prism 6.

To better understand the distribution of miRNAs expression in patients with periodontitis at different severity, we analyzed their expression levels based on disease stages. In detail, although an increase in expression levels was observed for miR-15a-5p and miR-223-3p in comparison with HC, there were no significant differences between disease stages (Fig. 2a, b). The expression levels of miR-23a-3pshowed a fold change of 11.32 in patients with mild periodontitis compared to HC, while, in moderate and severe periodontal disease, the expression levels were 1.9 and 1.4-fold respectively. Furthermore, in our patients, miR-23a-3p was significantly increased in mild periodontitis patients also in comparison with moderate and severe periodontitis patients (p < 0.001) (Fig. 2c).

About miR-103a-3p, increasing expression levels were observed in relation to the different disease stages, with significant differences between mild and severe stages (p = 0.023), with a variation of 1.27-fold (Fig. 2d). Similarly, miR-423-5p showed an up-regulated expression in relation to disease severity, with a significant increase in severe periodontal disease patients, equal to 3.68-fold, in relation to patients with periodontitis in mild (0.39-fold) and moderate stage (0.45-fold) (Fig. 2e).

**Figure 3:**
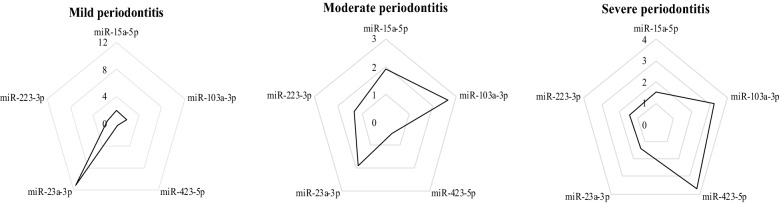
Radar plot of relative miRNAs expression profiles according to periodontitis stages.

The distribution of differential miRNAs expression was confirmed by the radar plot analysis. As shown in Fig. 3, in patients with mild periodontitis, a higher distribution area was reported for miR-23a-3p, supporting the involvement in the early phases of periodontal disease. Moreover, with a lower join area, miR-23a-3p was present also in patients with moderate disease. Furthermore, a major distribution of miR-103a-3p was observed in patients with moderate and severe periodontitis, while in patients with severe disease, we observed a higher distribution of miR-423-5p.

### Correlation analysis of miRNAs expression levels and inflammatory mediators

The analysis of the correlation of miRNAs with inflammatory responses in patients with periodontitis pointed out that the cytokines levels were significantly correlated between them and with the disease stage, confirming the increased release in parallel with the disease worsening. Results shown in Table 4 indicate a positive correlation between GCF levels of miR-103a-3p and TNFα in patients with moderate (r = 0.418, p < 0.001) and severe (r = 0.555, p < 0.01) periodontal disease. Moreover, we observed a positive correlation between miR-423-5p and cytokines in patients at severe disease stage (TNFα: r = 0.584, p < 0.01; IL-6: r = 0.526, p < 0.05), in accordance with the up-regulated miRNAs expression and higher inflammatory cytokines levels. Moreover, a negative correlation between miR-23a-3p and TNFα (r = -0.891, p < 0.01) and IL-6 (r = − 0.503, p < 0.05) was observed in the early stage of periodontal disease (Table 6).

**Table 6:** Analysis of the correlation of miRNAs expression levels with TNFα and IL-6.

Variable	TNFα (pg/ml)			IL-6 (pg/ml)		
	Mild	Moderate	Severe	Mild	Moderate	Severe
TNFα (pg/ml)	–	–	–	0.656***	0.568**	0.420**
IL-6 (pg/ml)	0.656***	0.568**	0.420**	–	–	–
miR-103a-3p	− 0.159	0.418***	0.555**	− 0.126	0.262	0.422
miR-423-5p	− 0.017	− 0.071	0.584***	0.115	0.092	0.526*
miR-15a-5p	0.021	0.240	0.366	0.049	0.512	0.573
miR-23a-3p	− 0.891***	− 0.099	0.060	− 0.503*	− 0.129	0.127
miR-223-3p	− 0.178	0.492*	0.112	− 0.147	0.339	0.279

The statistical significance shown are *p < 0.05; **p < 0.01; ***p < 0.001. Data were analyzed using Stata version 15.

Finally, genes were retrieved by GeneCards (https://www.genecards.org/) database^27^ using “inflammation and bone metabolism” as keyword. As reported in Table 7 the intersection of TNFα, IL-6, miR-103a-3p, miR-423-5p, miR-23a-3p, miR-15a-5p and miR-223-3p were portrayed by the GeneCards Inferred Functionality Score and the relevance score.

**Table 7:** GeneCards’ search.

Symbol	Description	Category	GIFtS	GC id	Score
IL6	Interleukin 6	Protein Coding	58	GC07P022725	91.57
TNF	Tumor Necrosis Factor	Protein Coding	59	GC06P092154	69.14
MIR223	MicroRNA 223	RNA Gene	25	GC0XP066018	31.98
MIR15A	MicroRNA 15a	RNA Gene	18	GC13M050049	20.97
MIR23A	MicroRNA 23a	RNA Gene	23	GC19M014657	20.49
MIR423	MicroRNA 423	RNA Gene	21	GC17P030117	9.75
MIR103A1	MicroRNA 103a-1	RNA Gene	20	GC05M168560	7.24

GIFsT, GeneCards Inferred Functionality Scores; GC id, GeneCards Identifiers; Score, Relevance Scores.

## Discussion

Periodontitis, one of the top ten highly prevalent diseases worldwide^28^, presents an amplified inflammatory immune response as a hallmark. The prevalent inflammatory microenvironment, affecting the periodontium and compromising tooth-supporting apparatuses (gingiva, cementum, periodontal ligament, and alveolar bone), negatively influences the general state of health, leading to the persistence of chronic low-grade inflammation for lengthened periods and increasing the risk for cardiovascular, cerebrovascular and respiratory diseases, with a higher probability for metabolic diseases development^29–31^. Much of the tissue damage that occurs during inflammation can be attributed to TNFα and IL-6. The balance of TNFα and IL-6 mirrors the degree of the inflammatory burden contributing to periodontal inflammation^32^.

TNFα, a pro-inflammatory cytokine released by macrophages, is known for its extensive role in periodontitis-mediated bone loss. Increased concentration of TNFα observed in periodontitis correlates closely with tissue destruction and immune response^33^*.* Meanwhile, IL-6, a multifunctional cytokine, has several biological activities, including B-lymphocyte differentiation, T-lymphocyte proliferation, and the stimulation of immunoglobulin (Ig) secretion by B-lymphocytes^34^. Particularly, IL-6 induces bone resorption by itself and in conjunction with other bone-resorbing agents^35^.

Our results showed that TNFα and IL-6 production levels were significantly increased in patients with periodontal disease in comparison with HC. Additionally, significant differences in TNFα and IL-6 levels were found between patients in accord with the mild, moderate, and severe periodontitis stages. These data agree with previous findings showing significantly elevated salivary concentrations of IL-6 and TNFα in periodontitis patients^36–41^ and suggest that IL-6 and TNFα GCF levels may have the potential to distinguish different phases of periodontitis.

The miRNAs play a role in the production of pro-inflammatory cytokines by both a “direct” and “indirect” modulation, via the targeting activators or repressors. MiRNAs in body fluids have been reported as high stable molecules with resistance to degradation, suggesting great potential as biomarkers, also because they represent one of the most abundant classes of molecules involved in the regulation of gene expression^16^.

Despite significant advances in miRNAs involvement in periodontal disease, the regulatory mechanisms are still to be investigated, also due to heterogeneity of the miRNAs’ expression.

Taking in mind that the inflammation in periodontal disease often results in alveolar bone loss and disruption of connective tissue homeostasis^42–44^, we have evaluated in GCF the expression of miR-15a-5p, miR-23a-3p, miR-223-3p, miR-103a-3p, and miR-423-5p, involved in the regulation of innate immune response, cell metabolism, and inflammation, in patients with periodontal disease at three different disease stages (mild, moderate and severe).

The expression of selected miRNAs was up-regulated in patients with respect to HC, in agreement with previous observations^12,45^.

In accord with its role as a positive regulator of the inflammatory microenvironment and negative regulator of osteogenic differentiation in various cell types^46,47^, we have observed a high expression for miR-23a-3p in mild periodontal disease, probably acting as a starter for the bone metabolism alteration. Furthermore, miR-103a-3p, related to inflammation and bone metabolism targeting IL-1, prostaglandin E2 (PGE2), and TNFα, was up-regulated in periodontal inflamed tissue disease^48,49^. In our patients, miR-103a-3p was increased mostly in the moderate disease level. About miR-423-5p, participating in osteoclast metabolisms^26^, a down-regulation in mild and moderate disease stages was observed with respect to HC, although an over-expression in severe disease stage patients was detected. These data are in accordance with miR-423-5p involvement in osteoclastogenesis, which represents a critical step in periodontal disease physiopathology^50^, characterized by an active inflammatory state, with high levels of TNFα, needed for osteoclastic cellular maturation. Furthermore, it has been observed that miR-423-5p has a better positive relation with TNFα and IL-6, with the ability in NFkB pathway up-regulation. Referring to miR-223-3p, Bauernfeind et al. suggested its antimicrobial effect against periodontal pathogens^51^ and regulatory role of inflammation through neutrophil recruitment in chronic inflammatory sites. miR-223-3p is also involved in the differentiation of several immune cells, particularly macrophages, by influencing their activation patterns^52^. Therefore, has been reported that miR-223-3p is up-regulated in periodontal disease, to regulate tissue homeostasis, to control osteoblast differentiation, and to avoid alveolar bone loss, which are emblems of periodontitis^53^. In our study groups, although a slight increase in miR-223-3p expression levels has been observed in periodontal disease patients in comparison with HC, no significant differences between the three disease stages were observed, demonstrating a non-decisive role in the regulation of disease progression. miR-15a-5p is reported to be closely related to several diseases, mediating the inflammatory process, but its role is not plenty investigated in periodontal disease. Luan et al. found the up-regulation of miR-15a-5p in both humans and mice affected by periodontitis^7^. In our patients, although not significantly, miR-15a-5p expression showed a reduced trend during disease progression in parallel with the increase of TNFα and IL-6 levels in GCF. Our results are encouraging, suggesting a different involvement of selected miRNAs in relation to the disease stages. The strong correlation found between miRNAs expression and inflammatory cytokine levels in relation to disease severity supports the deepening of their role as disease markers. Also, it is very interesting that this can be explored in GCF, supporting its high diagnostic, prognostic and therapeutic potential. However, their clinical application still needs to be defined and investigated.

## Materials and methods

### Patient selection and clinical periodontal parameter examination

Fifteen periodontally HC (aged 57–63 years) and twenty-one patients with periodontal disease (aged 56–61 years), all non-smokers, were referred to the Dental Clinic of the Department of Innovative Technologies in Medicine and Dentistry, “G. d’Annunzio” University, Chieti-Pescara, Italy, and participated in this study.

The inclusion criteria were as follows:

- Patients between 18 and 75 years
- Patients with periodontal disease
- Need of periodontal therapy
  The exclusion criteria were as follows:
- Poor oral hygiene
- Uncontrolled diabetes mellitus
- Immune diseases
- Smoking
- Bruxism

Every patient and healthy individual enrolled in the investigation provided written informed consent. In addition, the research related to human use has complied with all the relevant national regulations, in accordance with the tenets of the Helsinki Declaration, and has been approved by the experimental research ethics committee of the “G. d’Annunzio” University (254/2019). Diagnosis and classification of the periodontal disease were done according to the new classification, defined in the context of the 2017 World Workshop^3^, based on clinical and radiographic data. CAL and MPD were measured as previously reported^54^, to define the disease stages in individual patients. Healthy individuals assumed as the control group did not present any sign or symptom compatible with periodontal disease.

### Gingival crevicular fluid samples collection

The GCF sample was taken from a single-rooted tooth, due to its easy access and to avoid errors associated with GCF sample collection, both in patients with periodontitis and HC. Before placing the absorbing paper strip within the sulcus, the supragingival plaque was removed and relative isolation was performed using cotton rolls and aspiration to prevent saliva contamination. Subsequently, the filter paper strip Periopaper (Oraflow, New York, NY, USA) was placed in the gingival sulcus until resistance was felt and left in this position for 30 s following the routine procedure. The filter paper strip was introduced in a sterile tube and stored at − 80 °C, until the subsequent analysis. Meanwhile, the demographic and habit data of the patients enrolled in both groups have been collected on specific Case Report Forms.

### RNA extraction and quantification from gingival crevicular fluid

Periopapers were incubated in phosphate-saline buffer (PBS) solution pH 7 for 30 min at room temperature. To recover all the solution contained in the strip, each sample was centrifuged at 500 rpm for 10 min. Cell-free total RNA (including miRNAs) was isolated from 200 µL of PBS solution containing the biological material from the Periopaper using a miRNeasy Serum/Plasma kit (Qiagen, Hilden, Germany). RNA was eluted with 25 µL of RNAse-free water. Total RNA concentrations were quantified using NanoDrop ND 2000 UV-spectrophotometer (Thermo Scientific, Delaware, USA).

### Quantitation of miRNAs by real-time PCR

Reverse transcription (RT) reactions were performed using the miRCURY LNA RT Kit (QIAGEN, Hilden, Germany). Real-time PCR reactions were performed in triplicate, in 10µL reaction volumes, using miRCURY LNA SYBR Green PCR Kit (QIAGEN, Hilden, Germany). miRNAs gene expressions were evaluated for the following sequences:

hsa-miR-23a-3p: 5′AUCACAUUGCCAGGGAUUUCC.

hsa-miR-423-5p: 5′UGAGGGGCAGAGAGCGAGACUUU.

hsa-miR-15a-5p: 5′UAGCAGCACAUAAUGGUUUGUG.

hsa-miR-223-3p: 5′UGUCAGUUUGUCAAAUACCCCA.

has-miR-103a-3p: 5′AGCAGCAUUGUACAGGGCUAUGA.

UniSp6: 5′CUAGUCCGAUCUAAGUCUUCGA.

using UniSp6 as an endogenous control gene. Real-time PCR was carried out on CFX Real-Time PCR Detection Systems (Bio-Rad, Hercules, California, USA), programmed as follows: 50 °C for 2 min, 95 °C for 10 min followed by 45 cycles of 95 °C for 15 s and 60 °C for 1 min. The relative gene expression was calculated in the periodontal disease group vs the HC according to the formula 2^−∆∆Ct^^55^. The calculations were performed in MS Excel and graphs were drawn using GraphPad Prism 6 (GraphPad, La Jolla, CA, USA).

### ELISA assay

The human TNFα and IL-6 concentrations were measured by ‘sandwich’ ELISA (Millipore, Merck KGaA, Germany) on the GCF sample, according to the manufacturer’s instructions. The absorbance of each well was detected with a spectrophotometer, allowing for the generation of a standard curve and subsequent determination of protein concentration. The range of the standard curves was 1.37–12,000 pg/mL for IL-6 and 24.58–6000 pg/mL for TNFα.

### Statistical analysis

The mean was taken as the measurement of the main tendency, and the SD was taken as the dispersion measurement for the statistical analysis of the results. Qualitative variables were reported as frequency and percentage. The normal distribution of data was tested by the Kolmogorov–Smirnov test. Student’s t-test was performed to compare baseline variables between study groups. Radar plot analysis for the distribution of differential expression patterns of miRNAs was evaluated. Pearson’s correlation analysis was calculated to assess the relationship between cytokine levels and miRNAs gene expression in patients. Statistically significant was set at p-value < 0.05. Data analysis was performed with Stata version 15 (StataCorp LLC, College Station, TX, USA) and GraphPad Prism 6 Software, version 6.01, 2012.

### Ethical approval and consent to participate

This study conformed to the provisions of the Declaration of Helsinki. The protocol was approved by the Ethics Review Committee of G.d’Annunzio University, Chieti-Pescara (Protocol code 254/2019). Informed consent was obtained from all participants.

## Strengths and limitations of the study

Our data underline the importance of crevicular fluid as a possible diagnostic tool and means to monitor the status of patients affected by periodontal disease. On the other hand, the relatively small number of studied samples limits the possibility of generalizing the obtained considerations and of using these data to define the diagnostic sensitivity and specificity of the studied miRNAs. The evaluation of miRNAs could be accompanied by the analysis of the proteome, transcriptome and microbiome, in order to obtain a complete picture of their role in periodontal disease.

## Data Availability

The data supporting the results of this article are included within the article and can be required to the corresponding authors.
